# Editorial: Soft aerial robots: Design, control, and applications of morphologically adaptive flyers

**DOI:** 10.3389/frobt.2023.1196942

**Published:** 2023-04-21

**Authors:** Pham Huy Nguyen, Mirko Kovač, Begoña C. Arrue

**Affiliations:** ^1^ Aerial Robotics Lab, Imperial College London, London, United Kingdom; ^2^ Laboratory of Sustainability Robotics, Swiss Federal Laboratories for Material and Science (Empa), Dübendorf, Switzerland; ^3^ Grupo de Robotica, Visión y Control, Universidad de Sevilla, Sevilla, Spain

**Keywords:** soft roboitcs, aerial robotics, smart materials, manipulation, collision-resilance, physical artificial intelligence, embodied intelligence

In recent years, there has been a conscious effort in the field of aerial robots to trend towards developing flying vehicles that are more mechanically robust, maneuverable, energy efficient, capable of accessing multi-terrains and performing multiple functions. By combining fundamental knowledge of biological flyers with multidisciplinary sciences such as material science, biology, chemistry, computer science, and mechanical engineering, dawns a new age of aerial robots with interesting physical artificial intelligent characteristics, called soft aerial robots. This Research Topic aims to showcase high-quality work from leading researchers in the field. Through this Research Topic, the current state of the art, technical and conceptual barriers, and future opportunities for the topic of soft aerial robotics are discussed.

The selected papers in this issue highlights work ranging from quadrotors with flexible arms and rotor-tilting mechanisms in Ruiz et al. and Tang et al. Flapping aerial robots with embodied airflow sensing to improve in-gust flight in Wang et al. Interactive graspers to capture micro-aerial vehicles mid-air in Liu et al. And finally, biodegradable gliders inspired by plant seeds for environmental sensing in Wiesemüller et al.

In the paper, “*Aeroelastics-aware compensation system for soft aerial vehicle stabilization*,” Ruiz et al. describes a newly designed flexible arm, now controlled using a tendon-actuated system. This system is utilized for in-flight stabilization, further improving the energy-efficiency up to 30%, compared to its predecessor. The stabilization method models the aerodynamic interactions of the flexible arms, taking into account bending perturbations during pitching and rolling, and any torsion disturbances during yaw maneuvers. Furthermore it rejects aeroelastic perturbations utilizing the Ziegler-Nichols method. The work concludes that maneuvers are only possible up to angles of 60% of its range of motion. In the future, the team is looking to perform object grasping with multi-functional additional arms, among other flight performance improvements.

In the paper, “*QUaRTM: A Quadcopter with Unactuated Rotor Tilting Mechanism capable of faster, more agile, and more efficient flight*,” Tang et al. describes a new quadcopter design, capable of achieving two tilt configurations, mid-flight, by utilizing only the flight motors. To achieve this, the authors utilize sprung hinges instead of classical rigid connections. Tilting the configurations reduces the drag area, so the system can perform faster, more efficient and agile flights. The goal of the varying configuration is to switch between applications that need time-sensitive tasks, like package delivery or drone racing. The future goals of the project is to design the system for a wider range of flight performance through the choice of the sprung spring hinges and the redesign of the vehicle frame to be more aerodynamic.

In the paper, “*Safely catching aerial micro-robots in mid-air using an open-source aerial robot with soft gripper*,” Liu et al. describes an aerial robot equipped with a soft gripper to catch another aerial micro-robot mid-air. The Soft Aerial Gripper (SoAG) is an open-source, onboard gripper that is pneumatically driven. Furthermore, to avoid the downwash of the quadrotor, the SoAG is mounted for horizontal grasping. The system is tested to capture micro-robots with and without propeller guards, moving at an average velocity of 0.2 m/s. A planning method is developed based on piecewise polynomial optimization to catch the micro-robots, without generating any additional aerodynamic disturbances that would affect the micro-robot target’s stability. The eventual goal of the system is to be used in search and rescue of aerial robots, to capture small-scale unidentified flying targets without damaging the target. In the future, the authors plan to design a system capable of grasping and carrying larger payloads in a robust manner for outdoor expeditions. The authors also want to combine their soft aerial and ground robots together.

In the paper, “*Embodied airflow sensing for improved in-gust flight of flapping wing MAVs*,” Wang et al. explores the sensing of airflow in order to enable flapping wing micro aerial vehicles (FWMAVs) to operate in conditions with wind and gusts. The authors first generate a reliable model of the in-gust flight dynamics for the adaptive control framework, and then add the embodied airflow-sensing approach to see an improvement of 25.15% when subjected to frontal wind gusts, of up to 2.4 m/s. The controller also highlights improved energy efficiency. In the future, the authors aim to investigate adaptive controllers for the thrust control loop, in order to further improve position control and energy efficiency.

Finally, in the paper, “*Transient bio-inspired gliders with embodied humidity responsive actuators for environmental sensing*,” Wiesemüller et al. studied the gliding *Alsomitra macrocarpa* seed (Javan cucumber seed) in order to develop a library of fully transient and biodegradable aerial gliders. The design space of the gliders were studied and optimized based on the glider’s dihedral, airfoil camber, and asymmetric features. An embodied humidity actuator sits onboard the glider, as a payload that adjusts the CG for optimized flight performance. When it reacts to rainwater, the actuator opens, allowing an onboard pH sensor to be exposed to the rainwater. The system also highlights biodegradability, disintegrating completely within a week. To utilize these sensing gliders in the real-world, a quadcopter with a release mechanism is developed to deploy the gliders from a set height. In the future, the authors want to expand the library of biodegradable sensors onboard the gliders to measure temperature, UV intensity, micro pollutant levels, to name a few.

